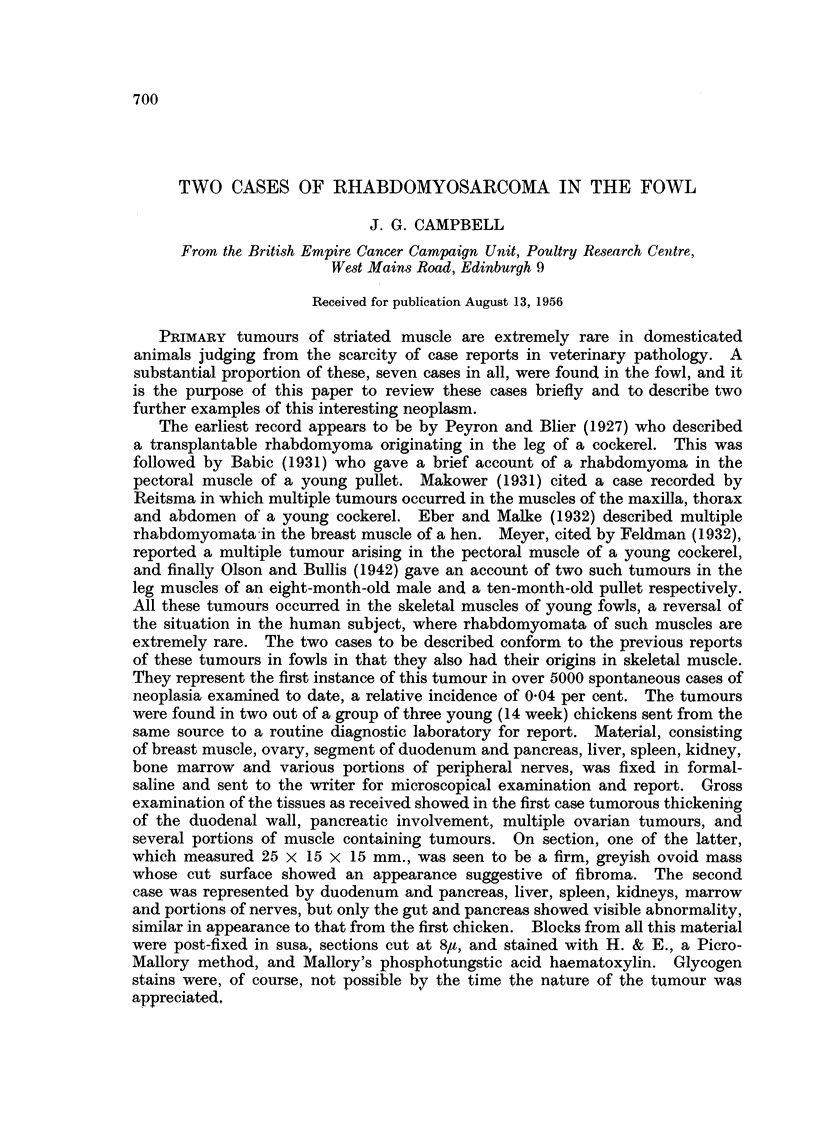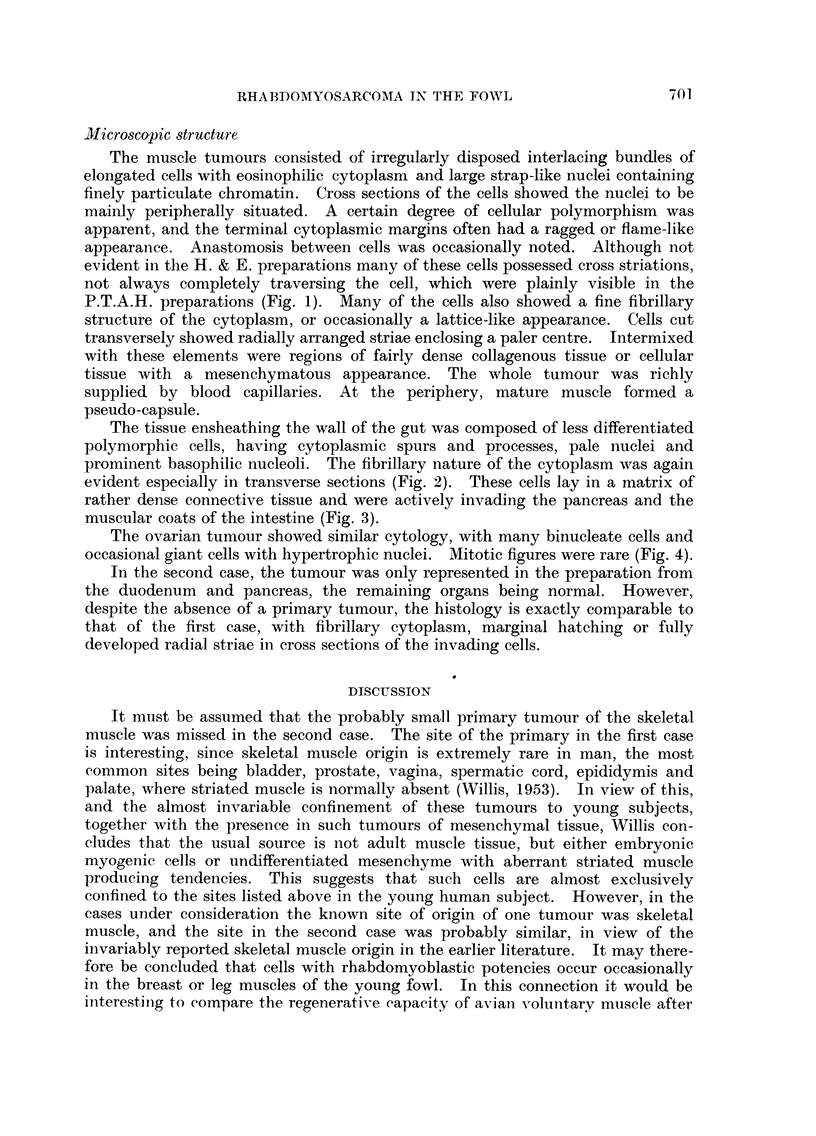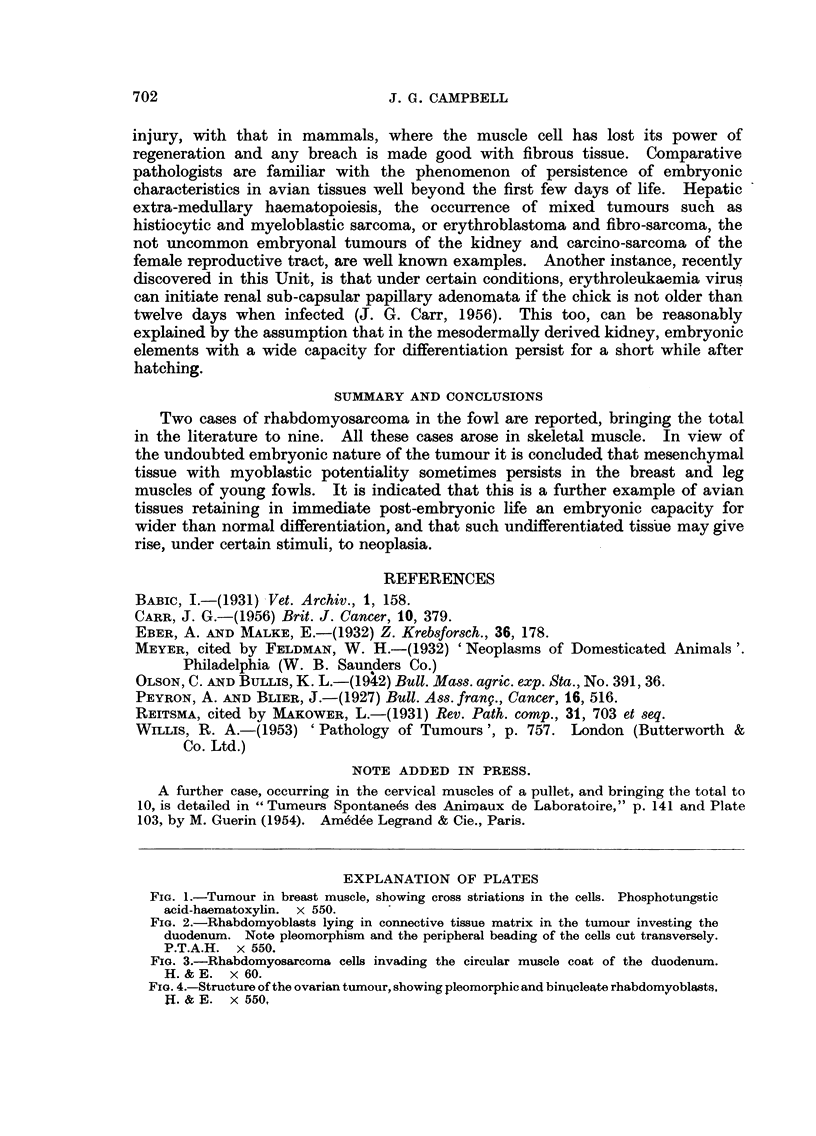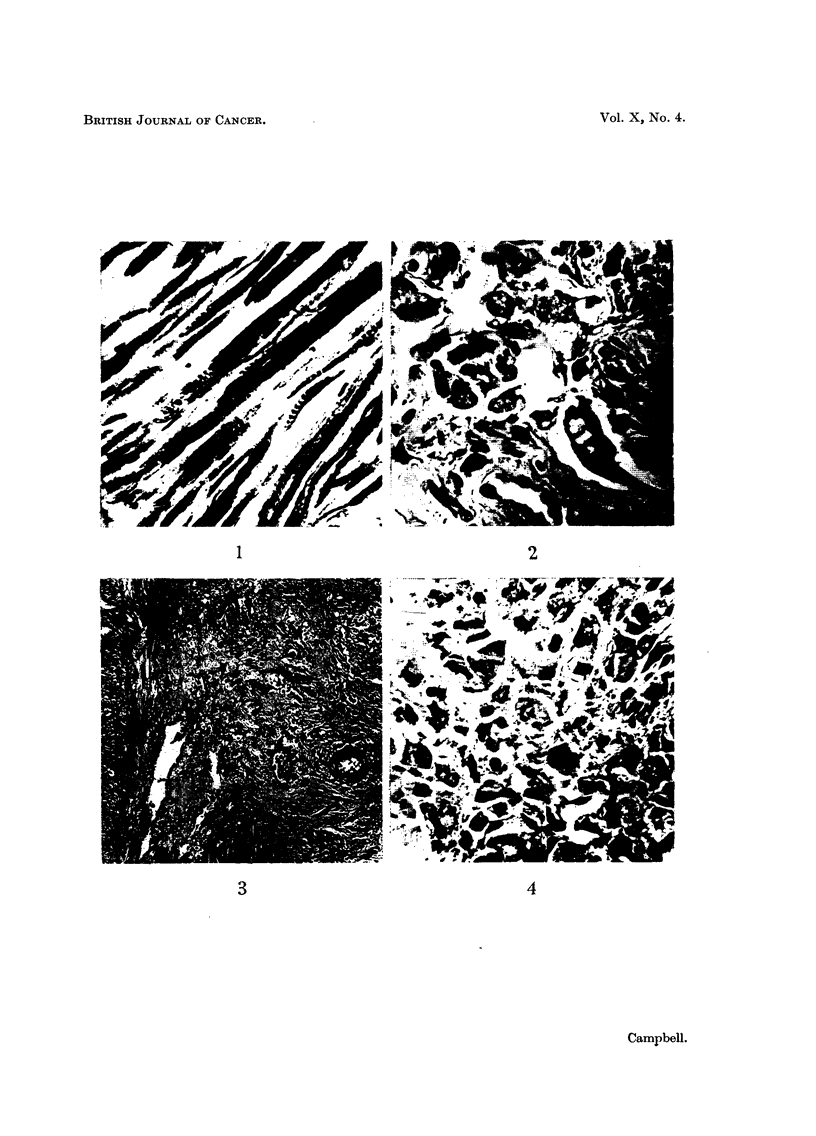# Two Cases of Rhabdomyosarcoma in the Fowl

**DOI:** 10.1038/bjc.1956.84

**Published:** 1956-12

**Authors:** J. G. Campbell

## Abstract

**Images:**


### TWO CASES OF RHABDOMYOSARCOMA IN THE FOWL

J. G. CAMPBELL

*From the British Empire Cancer Campaign Unit, Poultry Research Centre, West Mains Road, Edinburgh 9*

Received for publication August 13, 1956

PRIMARY tumours of striated muscle are extremely rare in domesticated animals judging from the scarcity of case reports in veterinary pathology. A substantial proportion of these, seven cases in all, were found in the fowl, and it is the purpose of this paper to review these cases briefly and to describe two further examples of this interesting neoplasm.

The earliest record appears to be by Peyron and Blier (1927) who described a transplantable rhabdomyoma originating in the leg of a cockerel. This was followed by Babic (1931) who gave a brief account of a rhabdomyoma in the pectoral muscle of a young pullet. Makower (1931) cited a case recorded by Reitsma in which multiple tumours occurred in the muscles of the maxilla, thorax and abdomen of a young cockerel. Eber and Malke (1932) described multiple rhabdomyomata in the breast muscle of a hen. Meyer, cited by Feldman (1932), reported a multiple tumour arising in the pectoral muscle of a young cockerel, and finally Olson and Bullis (1942) gave an account of two such tumours in the leg muscles of an eight-month-old male and a ten-month-old pullet respectively. All these tumours occurred in the skeletal muscles of young fowls, a reversal of the situation in the human subject, where rhabdomyomata of such muscles are extremely rare. The two cases to be described conform to the previous reports of these tumours in fowls in that they also had their origins in skeletal muscle. They represent the first instance of this tumour in over 5000 spontaneous cases of neoplasia examined to date, a relative incidence of 0·04 per cent. The tumours were found in two out of a group of three young (14 week) chickens sent from the same source to a routine diagnostic laboratory for report. Material, consisting of breast muscle, ovary, segment of duodenum and pancreas, liver, spleen, kidney, bone marrow and various portions of peripheral nerves, was fixed in formal-saline and sent to the writer for microscopical examination and report. Gross examination of the tissues as received showed in the first case tumorous thickening of the duodenal wall, pancreatic involvement, multiple ovarian tumours, and several portions of muscle containing tumours. On section, one of the latter, which measured $25 \times 15 \times 15$ mm., was seen to be a firm, greyish ovoid mass whose cut surface showed an appearance suggestive of fibroma. The second case was represented by duodenum and pancreas, liver, spleen, kidneys, marrow and portions of nerves, but only the gut and pancreas showed visible abnormality, similar in appearance to that from the first chicken. Blocks from all this material were post-fixed in susa, sections cut at $8\mu$, and stained with H. & E., a Picro-Mallory method, and Mallory's phosphotungstic acid haematoxylin. Glycogen stains were, of course, not possible by the time the nature of the tumour was appreciated.

*Microscopic structure*

The muscle tumours consisted of irregularly disposed interlacing bundles of elongated cells with eosinophilic cytoplasm and large strap-like nuclei containing finely particulate chromatin. Cross sections of the cells showed the nuclei to be mainly peripherally situated. A certain degree of cellular polymorphism was apparent, and the terminal cytoplasmic margins often had a ragged or flame-like appearance. Anastomosis between cells was occasionally noted. Although not evident in the H. & E. preparations many of these cells possessed cross striations, not always completely traversing the cell, which were plainly visible in the P.T.A.H. preparations (Fig. 1). Many of the cells also showed a fine fibrillary structure of the cytoplasm, or occasionally a lattice-like appearance. Cells cut transversely showed radially arranged striae enclosing a paler centre. Intermixed with these elements were regions of fairly dense collagenous tissue or cellular tissue with a mesenchymatous appearance. The whole tumour was richly supplied by blood capillaries. At the periphery, mature muscle formed a pseudo-capsule.

The tissue ensheathing the wall of the gut was composed of less differentiated polymorphic cells, having cytoplasmic spurs and processes, pale nuclei and prominent basophilic nucleoli. The fibrillary nature of the cytoplasm was again evident especially in transverse sections (Fig. 2). These cells lay in a matrix of rather dense connective tissue and were actively invading the pancreas and the muscular coats of the intestine (Fig. 3).

The ovarian tumour showed similar cytology, with many binucleate cells and occasional giant cells with hypertrophic nuclei. Mitotic figures were rare (Fig. 4).

In the second case, the tumour was only represented in the preparation from the duodenum and pancreas, the remaining organs being normal. However, despite the absence of a primary tumour, the histology is exactly comparable to that of the first case, with fibrillary cytoplasm, marginal hatching or fully developed radial striae in cross sections of the invading cells.

### DISCUSSION

It must be assumed that the probably small primary tumour of the skeletal muscle was missed in the second case. The site of the primary in the first case is interesting, since skeletal muscle origin is extremely rare in man, the most common sites being bladder, prostate, vagina, spermatic cord, epididymis and palate, where striated muscle is normally absent (Willis, 1953). In view of this, and the almost invariable confinement of these tumours to young subjects, together with the presence in such tumours of mesenchymal tissue, Willis concludes that the usual source is not adult muscle tissue, but either embryonic myogenic cells or undifferentiated mesenchyme with aberrant striated muscle producing tendencies. This suggests that such cells are almost exclusively confined to the sites listed above in the young human subject. However, in the cases under consideration the known site of origin of one tumour was skeletal muscle, and the site in the second case was probably similar, in view of the invariably reported skeletal muscle origin in the earlier literature. It may therefore be concluded that cells with rhabdomyoblastic potencies occur occasionally in the breast or leg muscles of the young fowl. In this connection it would be interesting to compare the regenerative capacity of avian voluntary muscle after injury, with that in mammals, where the muscle cell has lost its power of regeneration and any breach is made good with fibrous tissue. Comparative pathologists are familiar with the phenomenon of persistence of embryonic characteristics in avian tissues well beyond the first few days of life. Hepatic extra-medullary haematopoiesis, the occurrence of mixed tumours such as histiocytic and myeloblastic sarcoma, or erythroblastoma and fibro-sarcoma, the not uncommon embryonal tumours of the kidney and carcino-sarcoma of the female reproductive tract, are well known examples. Another instance, recently discovered in this Unit, is that under certain conditions, erythroleukaemia virus can initiate renal sub-capsular papillary adenomata if the chick is not older than twelve days when infected (J. G. Carr, 1956). This too, can be reasonably explained by the assumption that in the mesodermally derived kidney, embryonic elements with a wide capacity for differentiation persist for a short while after hatching.

### SUMMARY AND CONCLUSIONS

Two cases of rhabdomyosarcoma in the fowl are reported, bringing the total in the literature to nine. All these cases arose in skeletal muscle. In view of the undoubted embryonic nature of the tumour it is concluded that mesenchymal tissue with myoblastic potentiality sometimes persists in the breast and leg muscles of young fowls. It is indicated that this is a further example of avian tissues retaining in immediate post-embryonic life an embryonic capacity for wider than normal differentiation, and that such undifferentiated tissue may give rise, under certain stimuli, to neoplasia.

### REFERENCES

Babic, I.—(1931) *Vet. Archiv.*, **1**, 158.
Carr, J. G.—(1956) *Brit. J. Cancer*, **10**, 379.
Eber, A. and Malke, E.—(1932) *Z. Krebsforsch.*, **36**, 178.
Meyer, cited by Feldman, W. H.—(1932) 'Neoplasms of Domesticated Animals'. Philadelphia (W. B. Saunders Co.)
Olson, C. and Bullis, K. L.—(1942) *Bull. Mass. agric. exp. Sta.*, No. 391, 36.
Peyron, A. and Blier, J.—(1927) *Bull. Ass. franç., Cancer*, **16**, 516.
Reitsma, cited by Makower, L.—(1931) *Rev. Path. comp.*, **31**, 703 *et seq.*
Willis, R. A.—(1953) 'Pathology of Tumours', p. 757. London (Butterworth & Co. Ltd.)

### NOTE ADDED IN PRESS.

A further case, occurring in the cervical muscles of a pullet, and bringing the total to 10, is detailed in "Tumeurs Spontaneés des Animaux de Laboratoire," p. 141 and Plate 103, by M. Guerin (1954). Amédée Legrand & Cie., Paris.

---

### EXPLANATION OF PLATES

Fig. 1.—Tumour in breast muscle, showing cross striations in the cells. Phosphotungstic acid-haematoxylin. × 550.

Fig. 2.—Rhabdomyoblasts lying in connective tissue matrix in the tumour investing the duodenum. Note pleomorphism and the peripheral beading of the cells cut transversely. P.T.A.H. × 550.

Fig. 3.—Rhabdomyosarcoma cells invading the circular muscle coat of the duodenum. H. & E. × 60.

Fig. 4.—Structure of the ovarian tumour, showing pleomorphic and binucleate rhabdomyoblasts. H. & E. × 550.